# Chronic Pain and Mood Disorders in Asian Americans

**DOI:** 10.31372/20200504.1115

**Published:** 2021

**Authors:** Deborah L. Huang, Indraneil Bardhan, Joohyun Shin, Jordan F. Karp, Mijung Park

**Affiliations:** a University of Washington, Seattle, Washington, United States; b University of California, San Francisco, San Francisco, California, United States; c University of Pittsburgh, Pittsburgh, Pennsylvania, United States

**Keywords:** pain, depression, anxiety, mood disorder, comorbidity, Asian Americans

## Abstract

**Purpose:** Pain and mood disorder frequently coexist. Yet, for Asian Americans (AAs), scant information about pain and mood disorder is available. Our aims were to compare (1) the rates of pain and mood disorders and (2) the magnitude of associations between pain and mood disorders between AAs and European Americans (EAs), and across different Asian subgroups.

**Methods:** An analytical data was constructed from the Collaborative Psychiatric Epidemiology Studies (CPES), a representative sample of community-residing U.S. adults (*n* = 9,871). Pain morbidity was assessed by self-report. Mood disorders, including major depression and anxiety disorders, were assessed using the diagnostic interview. Analysis included descriptive statistics and multivariate logistic regression modeling. All analyses were weighted to approximate the U.S. populations, and controlled for sociodemographic and immigration characteristics.

**Results:** Greater proportion of EAs, compared to AAs, endorsed lifetime pain (56.8% vs. 35.8%). Having life pain disorders elevated the likelihood of lifetime mood disorder by more than 2-folds (weight adjusted odds ratio (WAOR): 2.12, 95% CI: 1.77, 2.55). Having pain disorders over the past 12 months elevated the likelihood of mood disorder in the same time period by more than 3-folds (WAOR: 3.29, 95% CI: 2.02, 5.37) among AAs. The magnitude of the association between pain and psychiatric morbidity were greater in Vietnamese Americans compared to other AAs and EAs.

**Discussion:** The conventional belief that rates of pain and mood disorders are greater in AAs than EAs may need to be further examined. Vietnamese Americans may be particularly vulnerable for experience of comorbid pain and mood disorders.

## Introduction

Chronic pain—defined as pain that lasts or recurs for longer than 3 months—is a significant source of human suffering and commonly seen in primary care settings (Treede et al., 2015). The prevalence of chronic pain among adults seen in the primary care setting ranged between 10% and 15% (Nahin, 2015). Mood disorders, including depression and anxiety disorders, are also common health issues in primary care settings (Gold et al., 2020; Strand, Castro-Marrero, Helland, Alegre, & Mengshoel, 2020). Up to 14% of patients seen in primary care have estimated to have depression (Gonzalez et al., 2010).

Pain and mood disorders frequently co-occur. About 50–60% of people treated for depression also report problems with pain (Narasimhan & Campbell, 2010). Co-existing pain and mood disorders leads to negative outcomes related to health and quality of life, including poorer prognoses of pain and mood disorders, elevated economic burdens, and increased morbidity and mortality (Armbrecht et al., 2020; Bragazzi et al., 2019; Sambamoorthi, Shah, & Zhao, 2017). Bidirectional relations between chronic pain and mood disorders are well documented. In other words, changes in pain severity are associated with changes in depression symptom severity, and vice versa (Gerrits, van Oppen, van Marwijk, Penninx, & van der Horst, 2014; Kroenke et al., 2011). The rates of pain and mood disorders are known to be associated with age (Kivrak, Kose-Ozlece, Ustundag, & Asoglu, 2016), education (Chang-Quan, Zheng-Rong, Yong-Hong, Yi-Zhou, & Qing-Xiu, 2010; Sj⊘gren, Ekholm, Peuckmann, & Gr⊘nbæk, 2009), gender (Calvó-Perxas, Vilalta-Franch, Turró-Garriga, López-Pousa, & Garre-Olmo, 2016), marital status (Bj⊘rnnes et al., 2018), and immigration status (Joshua Breslau & Chang, 2006; Soares & Jablonska, 2004) and economic status (Dorner et al., 2011; Thomtén, Soares, & Sundin, 2012). In summary, the rates of pain and mood disorders tend to increase among older adults, women, immigrants, and individuals with less education and less economic power, compared to their counterparts.

Additionally, past studies show significant racial and ethnic variations in the experience of pain and in the association between pain and mood disorders (Chan, Hamamura, & Janschewitz, 2013; Edwards, Moric, Husfeldt, Buvanendran, & Ivankovich, 2005; Green, Anderson et al., 2003; Hardt, Jacobsen, Goldberg, Nickel, & Buchwald, 2008). Many existing studies were conducted during decades ago and did not include Asian Americans (AAs). Therefore, whether ethnic variations associated with pain and mood disorder exist among AA subgroups remains underexamined.

Although AAs are one of the fastest growing minority populations (U.S. Census Bureau, 2007, 2011), only limited information is available to examine if AAs experience pain and mood disorders at similar rates with European Americans (EAs). Furthermore, AA is a heterogeneous group that consists of at least 43 different ethnic groups with unique cultural beliefs and practices, and with divergent history. These variations may explain individual differences in experience of physical and emotional pain and in preference for treatment modalities. Thus, elucidating similarities and differences in pain in the context of mental health issues among diverse Asian subgroups may improve our ability to design and provide more individualized health care for this vulnerable population. Yet, limited information is available to explain how cultural and historical contexts may be associated with pain and mental health outcomes in this population. Past studies often include very small numbers of AAs or aggregate them into “other” categories, making it impossible to examine more nuanced study of AAs.

Our paper addresses this knowledge gap using a nationally representative epidemiological data. Aims of this study were to (1) compare the rates of pain and mood disorders between AAs and EAs and (2) examine if the association between pain and mood disorders vary across different Asian subgroups and between AAs and EAs.

## Methods

### Sample

This is a cross-sectional, descriptive study, using publicly available, deidentified, un-coded data from the Collaborative Psychiatric Epidemiology Studies (CPES) (Alegria, Jackson, Kessler, & Takeuchi, 2007; Heeringa et al., 2004). Briefly, the purposes of CPES were to examine the prevalence, correlates, and clinical significance of psychiatric illnesses and to study patterns and correlates of treatment and treatment adequacy (Alegria et al., 2007). Sample was identified using multistage probability sampling methods and included community-residing adults (age 18 and over) in the United States. Although the CPES were completed in 2003, its data includes the only nationally representative epidemiological data of Asian American mental health. Therefore, we justify our choice of using CPES to pursue the study aims, described in the previous section.

The analytical data set for this study included deidentified information of 9,871 community-residing individuals. Among them, 7,587 were of European origin and 2,284 were of Asian origin (520 Vietnamese, 508 Filipino, 600 Chinese, and 656 Other Asians). The Other Asian group included Japanese, Korean, Asian Indian, and those who interviewed in English. Unfortunately, CPES did not disaggregate The Other Asian group (Alegria et al., 2007). Survey instruments were administered in the respondent’s choice of language, including English, Spanish, Chinese, Vietnamese, or Tagalog by trained bilingual lay interviewers. The demographic characteristics of Asian sample have been described extensively elsewhere (Park, Unützer, & Grembowski, 2013; Takeuchi et al., 2007). A majority of sample were female (53%) and married (69.1%). A majority of Asian respondents were foreign born (76.0%). As mentioned previously, this study includes publicly available, deidentified, noncoded data, which does not constitute human subjects research as defined at 45.CFR 46.102, and therefore, does not require IRB review.

### Assessment of Pain Disorders

We generated binary variables for lifetime chronic pain, 12-month chronic pain, and 12-month disabling pain based on the following three sequential questions. Data on lifetime pain were available for the entire sample. Twelve-month pain data were available for the AA sample only.

Participants were asked: “Have you ever had any of the following: arthritis or rheumatism, chronic back or neck problems, frequent or severe headache, or any other chronic pain.” Respondents were also asked about medically unexplained chronic pain, defined as “pain lasting six months or longer that is severe enough either to interfere a lot with normal activities or to cause a lot of emotional distress and that a doctor cannot find a physical cause to explain.” When a respondent answered yes to any of these pain conditions, lifetime chronic pain was coded 1. Those who endorsed lifetime chronic pain were asked: “During the past 12 months, did you still have arthritis or rheumatism, chronic back or neck problems, frequent or severe headache, any other chronic pains, or medically unexplained chronic pain?” When a respondent answered yes, 12-month chronic pain was coded 1. Refused or do not know was coded as missing.

### Assessment of Mood Disorders

Mood disorders included major depression and anxiety disorders. Life-time and 12-month mood disorders were identified using the diagnostic interview of the World Mental Health Survey initiative version of the World Health Organization Mental Health Service (WMH-CIDI) (Kessler & Üstün, 2004). Previous studies have documented good concordance between DSM-IV diagnoses based on CIDI and the structural clinical interview for DSM-IV (Kessler et al., 2004; Kessler et al., 2009). Anxiety disorders included agoraphobia without panic disorder, agoraphobia with panic disorder, generalized anxiety disorder, panic attack, panic disorder, posttraumatic stress disorder, adult separation anxiety disorder, and social phobia.

### Covariates

We selected covariates known to be associated with both rates of pain and of psychiatric illness in the previous section. These covariates included age, gender, marital status, education, nativity (US-born vs. non-US-born), and living in poverty. Living in poverty was based on the income reported by the respondents. Income-to-need ratio less than 1 at the household level poverty threshold defined by U.S. Census Bureau was considered living in poverty. For example, in 2003, a household income less than $18,400 for a family size of 4 would be considered living in poverty.

### Data Analysis

We first examined demographic, immigration, and clinical characteristics of the study sample by calculating weighted prevalence and 95% confidence intervals (CI). To examine the adjusted association between chronic pain and mood disorder, we used multivariate logistic regression models. To test modifying effects of ethnicity, we added the interaction terms (pain × ethnicity) to the logistic models. *F*-tests and adjusted Wald tests were used to test group differences.

All statistical estimates were weighted, using the recommended sampling weights, to account for individual-level unequal probabilities of selection into the samples, nonresponse rate, and additional poststratification to ensure representation of the U.S. population. We employed survey bootstrap resampling approach for variance estimation. All analyses were performed using Stata version 16, a statistical package (StataCorp, 2019).

## Results

Table 1 describes the characteristics of the study sample. The mean age of EAs was significantly greater than AAs. Compared to AAs, a greater proportion of EAs were divorced/separated/widowed and experienced greater rates of lifetime mood disorders and lifetime pain disorders. As mentioned earlier, information about 12-month pain morbidity was not available for EAs.

**Table 1:** Demographic Characteristics of Asian Americans and European Americans

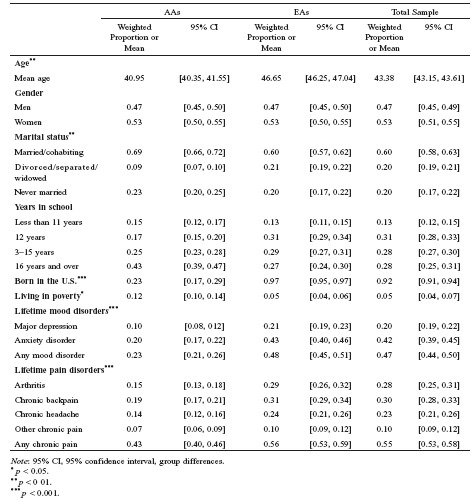

Table 2 provides detailed sample profiles of four AAs subgroups: Vietnamese, Filipino, Chinese, and All Other Asian. Compared to other AA subgroups, a greater proportion of Vietnamese sample lived in poverty, had less education, and born outside of United.States. Filipinos reported greater rates of life-time pain morbidity. We did not find statistically significant differences in other demographic and other clinical characteristics across AA subgroups.

**Table 2:** Demographic Characteristics of Asian American Subgroups

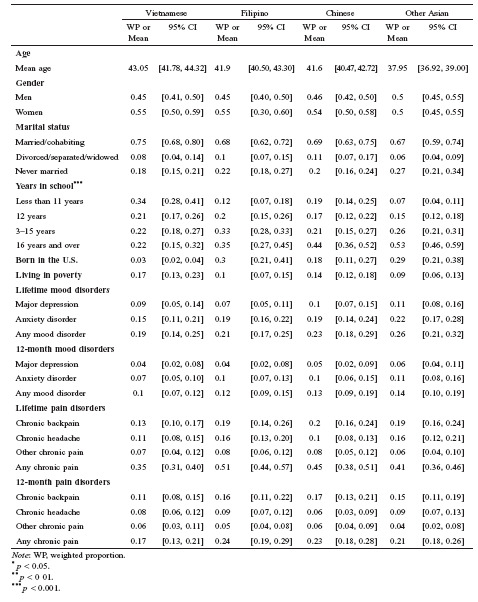

**Figure 1:**
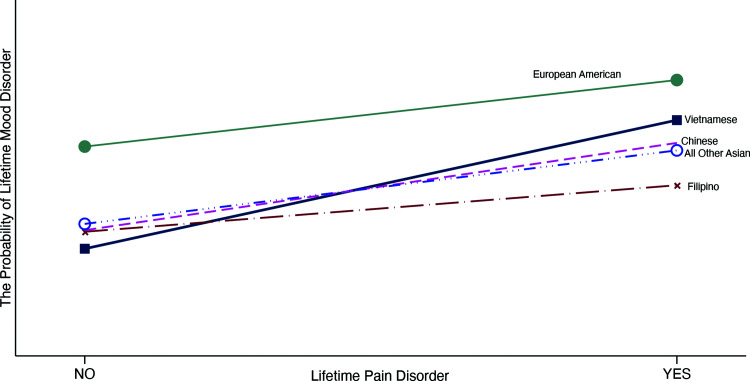
Differential magnitudes of the association between pain and mood disorders.

Table 3 summarizes the weighted and adjusted associations between lifetime chronic pain and lifetime mood disorders among different EA and AAs. Overall, having lifetime chronic pain was associated with about 2-fold increase in odds for having mood disorder over the same period of time [weight adjusted odds ratio (WAOR): 2.12, 95% CI: 1.77, 2.55]. However, we observed differential magnitudes in such association over the lifetime across different race/ethnic group. Compared to other groups, for example, having lifetime pain disorders increased the likelihood of lifetime mood disorders by more than 6.35-fold among Vietnamese Americans. However, having lifetime pain disorders increased the likelihood of lifetime mood disorders by about 2-fold. Figure 1 illustrates these differential magnitudes of the association between pain and mood disorders. For example, the likelihood of lifetime mood disorders was lower among Vietnamese Americans, compared to other Asian subgroups. However, among AAs with lifetime pain disorders, Vietnamese Americans experienced the greater likelihood compared to their other Asian counterparts.

**Table 3:** Differential Magnitude of the Association between Pain and Mood Disorders over the Lifetime

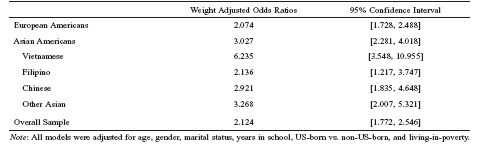

**Figure 2:**
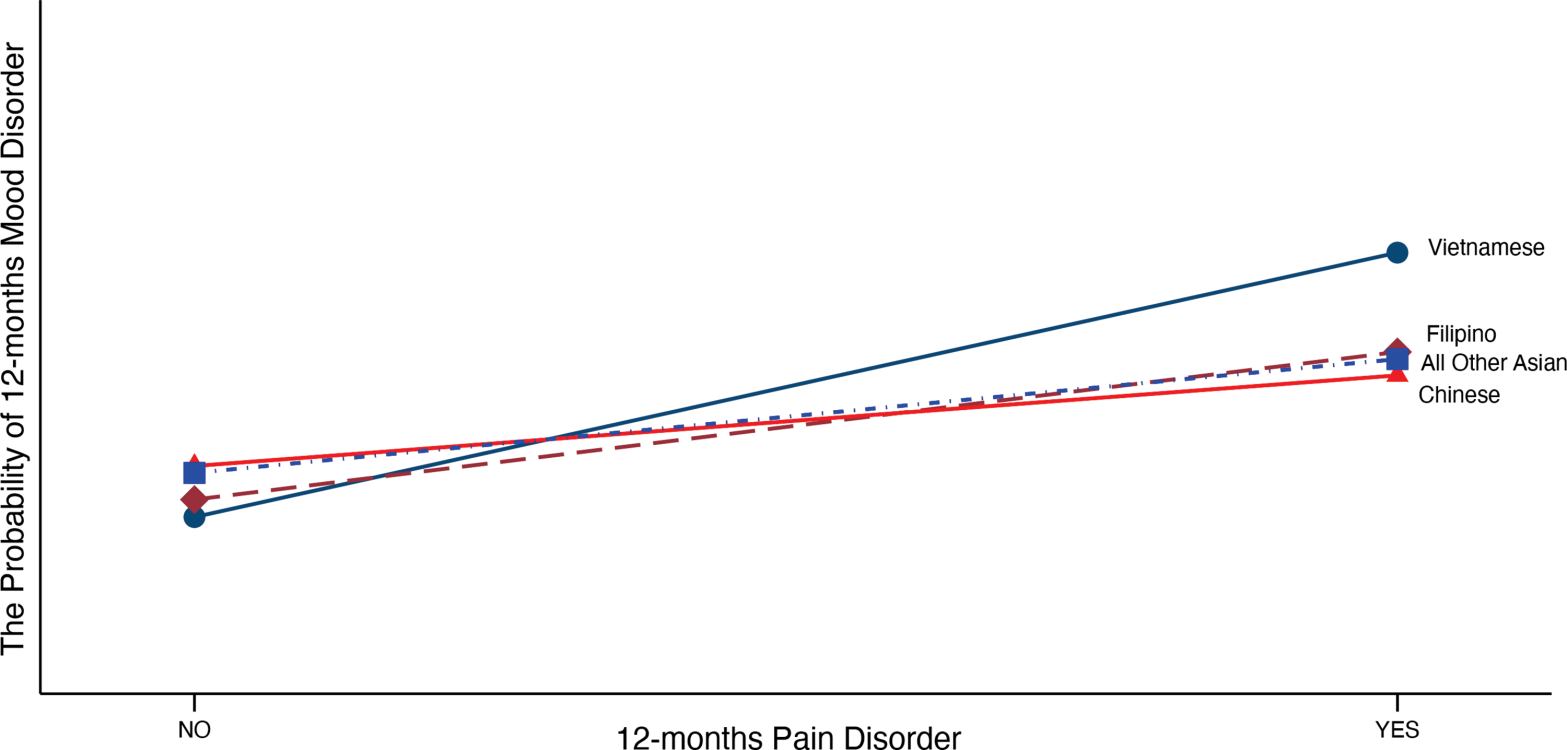
Differential magnitudes of the association between pain and mood disorders across AA subgroups.

Table 4 and Figure 2 describe weighted, adjusted associations between pain morbidity and psychiatric morbidity over the past 12 months. Having 12-month chronic pain was associated with more than 3-fold increase in the odds for having mood disorders over the same period of time in AAs (WAOR: 3.29, 95% CI: 2.02, 5.37). The magnitude of such association was greater among Vietnamese Americans compared to other AA subgroups.

**Table 4:** Differential Magnitude of the Association between Pain and Mood Disorders over the Past 12 Months

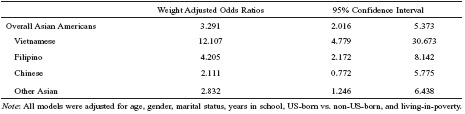

## Discussion

Our first aim was to compare the rates of pain and mood disorders between AAs and EAs. At the time of survey, the rates of lifetime pain and mood disorders were significantly greater among EAs, compared to their Vietnamese, Chinese, Filipino, and all other Asian counterparts. This finding is surprising because we expected that AAs would experience greater rates of mood disorders as well as pain morbidities. Existing literature have suggested that minorities experience greater burdens of psychiatric morbidities on pain outcomes than EAs (Green, Baker, Sato, Washington, & Smith, 2003; Green, Baker, Smith, & Sato, 2003; Miller & Cano, 2009). Moreover, findings of our study are also contradictory to the expectations based on the laboratory studies. Laboratory studies have reported that Asians tend to have lower pain tolerance and higher pain sensitivity (Campbell, Edwards, & Fillingim, 2005; Chan et al., 2013). Limited information exists to explain why AAs endorsed lower rates of pain and mood disorders over lifetime. Asian culture, which tends to encourage stoicism and discourage public expression of pain suffering may be one reason. Nevertheless, more studies are needed to gain more insights to explain why AA populations report lower rates of pain and mood disorders compared to EAs. As mentioned previously, direct comparison of 12-month pain and mood disorders between EAs and AAs was not possible due to the lack of data in EAs sample.

Our second aim was to compare the magnitude of associations between pain and mood disorders between AAs and EAs, and across AA subgroups. First, we found that having lifetime pain disorders was associated with more than 2-fold increase in the odds for lifetime mood disorders. This is consistent with consensus in the field that physical pain increases risk for emotional pain, and vice versa. The difference in the magnitudes of the association between lifetime pain and mood disorders between AAs as an aggregated group and EAs was not significant. Additionally, having 12-month chronic pain increased the likelihood of mood disorder by 3-fold among AAs as an aggregated group. The findings of our study of AAs are similar to those of the past analyses of representative sample of U.S. population (McWilliams, Cox, & Enns, 2003) and of California residents (Ohayon & Schatzberg, 2010); having current pain conditions is associated with increased odds, range between 1.48- and 3.86-fold, for having current psychiatric morbidity. In both studies, AAs were included in the “other” category.

Second, when we disaggregate AAs, we found that Vietnamese Americans experience greater magnitude of association between pain and mood disorder compared to EAs and to other AA subgroups. Our findings highlight that physical and emotional pain may be experienced differently by different ethnic group. For example, it may be plausible that Vietnamese Americans experience greater association between pain and mood disorders due to greater exposure to trauma compared to other ethnic groups. Thus, the ethnic variation we observed in this study may be related to their collective traumatic experience. Further research is needed to examine underlying mechanisms of subgroup variations in prevalence of pain and mood disorders and in the magnitude of the association between pain and mood. We also need more in-depth examination about how such information should be applied in design and implementation of health care services.

There are several limitations in this study. First, due to the cross-sectional nature of this study, we are unable to establish causal inference. Second, because information about pain morbidities is collected solely via respondents’ recall, the overall rates of painful conditions, especially lifetime rates of chronic pain, may have been underestimated. Third, although medically unexplained pain was clearly defined for the respondents, the chronicity of other pain conditions was not clearly defined. The respondents were asked if they had “chronic pain” in lifetime or in past 12 months and each respondent might have defined chronicity of their pain differently. Thus, we caution readers when comparing the findings of our study with other studies of chronic pain. Fourth, the “other” Asian group was an aggregated sample of all other Asian ethnic groups. While Vietnamese American, Filipino American, and Chinese American samples were offered culturally and linguistically appropriate surveys (Alegria et al., 2004), such an option was not provided to the “other” Asian sample. Therefore, we are unable to interpret the results of the Other Asian subgroup in a meaningful way. Finally, the available data on pain was not uniform across the three CPES studies, limiting our ability to directly compare the 12-month pain and mood disorders between EAs and AAs.

Despite these limitations, our paper contributes to the literature in several ways. First, this is among the first studies describing the prevalence of diverse pain conditions in AA populations using a representative sample of community-dwelling adults. Thanks to the design of CPES studies that allow pooling, we were able to directly compare the lifetime prevalence of pain and the associations between pain and psychiatric morbidity between AAs and EAs. Second, we used matching time references for the variables of interest. In other words, the risk of lifetime mood disorders was examined in the context of lifetime pain morbidities and the risk of 12-month mood disorders were examined in the context of 12-month pain morbidities. Third, we found little evidence for significant gender variations in the associations between 12-month pain and psychiatric morbidities among three major AAs. Nonetheless, certain Asian Americans (i.e., Vietnamese Americans) may be at greater risk for lifetime pain and psychiatric comorbidity.

## Conclusion

Having chronic pain significantly elevated the likelihood of mood disorders. AAs have lower rates of lifetime chronic and disabling pain than EAs. However, when having pain, AAs experience greater magnitude of elevated risks for having mood disorders, compared to EAs. Vietnamese Americans had significantly stronger associations between pain disorders and mood disorders during lifetime and in the past 12 months, compared to other Asian subgroups. Clinicians who frequently serve AA populations need to pay closer attention for patients who complain pain or mood issues and assess for comorbidity of pain and mood disorder. Furthermore, targeted public education about comorbidity of pain and mood disorder may be beneficial to improving public health and quality of life among AAs.

## Acknowledgment

The authors thank Alon Axelrod at the Inter-University Consortium for Political and Social Research (ICPSR) at the University of Michigan, for his valuable advice for weighting and resampling techniques.

## Declaration of Conflicting Interests

The authors declare that they have no financial interest or benefits arising from the direct applications of this study.

## Funding

This work was supported by the National Institute of Health (K01NR0515101 and P30 MH90333).
